# Transition of young people from CAMHS to AMH 2017-19

**DOI:** 10.1192/bjo.2021.465

**Published:** 2021-06-18

**Authors:** Vesna Acovski, Rahat Ghafoor, Rachel Shead

**Affiliations:** Leicestershire partnership NHS Trust

## Abstract

**Aims:**

Transition from CAMHS to AMH is recognised as a potential struggle for young people who suffer with poor mental health. In response to the 2017-19 NHS CQUIN project, LPT organised a monthly working group to establish the best transition process & deliver the CQUIN project.

**Background:**

It is estimated that more than 25,000 young people transition each year. It is reported that this process is often handled poorly, which can result in repeat assessments and emergency admissions for this large cohort of service users at a critical stage in life. The result is that young may go on to develop more severe problems in the absence of an appropriate transition service.

**Method:**

Cohort of service users eligible for transition (17yrs 6months) was identified. They were referred from CAMHS to AMH with a transition plan and referral letter. A face-to-face transition meeting was arranged which included the patient, carer & clinicians from sending & receiving services. A clinical audit was completed to ensure that care was transferred to AMH post-18th birthday of the patient. The process was followed up by pre- and post-transitions surveys.

**Result:**

From 110 identified service users 46% had joint-agency transition meeting and 79% had transition plan in place. 72% felt prepared to transition to AMH and 89% felt their transition goals were met. Positive comments have been received from service users.

**Conclusion:**

Link workers were identified to facilitate the transition process. Flow chart was established and disseminated across LPT. Services that need an improvement will be targeted and monitored. LPT will host an event for patients and carers to involve them in enhancing the transition process.

